# Characterization of Beta-Lactamases in Bloodstream-Infection *Escherichia coli*: Dissemination of bla_ADC__–__162_ and bla_CMY–__2_ Among Bacteria via an IncF Plasmid

**DOI:** 10.3389/fmicb.2019.02175

**Published:** 2019-10-01

**Authors:** Linlin Xiao, Xiaotong Wang, Nana Kong, Long Zhang, Mei Cao, Muzhen Sun, Quhao Wei, Weiwei Liu

**Affiliations:** ^1^Department of Laboratory Medicine, Affiliated Sixth People’s Hospital South Campus, Shanghai University of Medicine & Health Sciences, Shanghai, China; ^2^Department of Laboratory Medicine, Southern Medical University Affiliated Fengxian Hospital, Shanghai, China; ^3^Department of Laboratory Medicine, Affiliated Fengxian Hospital, Anhui University of Science and Technology, Anhui, China; ^4^Central Laboratory, Department of Laboratory Medicine, Shanghai Tenth People’s Hospital, Tongji University, Shanghai, China; ^5^Department of Laboratory Medicine, Shanghai Skin Disease Hospital, Tongji University, Shanghai, China

**Keywords:** beta lactamase, bloodstream infection, *Escherichia coli*, extraintestinal pathogenic, AmpC

## Abstract

**Objectives:**

To describe the molecular characteristics of beta-lactamases in bloodstream-infection *Escherichia coli* isolated from elderly patients, and to determine the genotypic patterns of *bla*_CMY__–__2_ and *bla*_ADC__–__162_.

**Methods:**

A total of 50 bloodstream-infection *E. coli* isolates were obtained from patients aged > 50 years at Shanghai Sixth People’s Hospital South Campus during 2015–2018. The isolates were subjected to beta-lactamase detection using phenotypic and molecular methods. Beta-lactamase genes were verified by sequencing and the phylogenetic relationships of the isolates were analyzed by multilocus sequence typing (MLST). The transferability of plasmids carrying *bla*_CMY–__2_ and *bla*_ADC–__162_ genes was verified by conjugation experiments and plasmid replicon typing.

**Results:**

Eight beta-lactamase subtypes were detected in 50 isolates of bloodstream-infection *E. coli*. *bla*_TEM–__1_ (21/50) was the most common beta-lactamase gene, followed by *bla*_CTX–M–__14_ (8/50), *bla*_OXA–__27_ (5/50), *bla*_CTX–M–__27_ (3/50), *bla*_CTX–M–__65_ (1/50), *bla*_ADC–__162_ (1/50), and *bla*_CMY–__2_ (1/50). Of these, *bla*_ADC–__162_ (ST95-A), and *bla*_CMY–__2_ (ST95-B2) have not previously been reported in bloodstream-infection *E. coli*. In 21 isolates, beta-lactamase genes were located on conjugative plasmids belonging to incompatibility groups FrepB (*n* = 7), FIA (*n* = 1), FIC (*n* = 2), K (*n* = 8), N (*n* = 1), and I (*n* = 1), and *bla*_CTX–M_ was associated with the common elements IS*Ecp*1, IS903, and IS26, but with special sequences (region V, region Y, and region W) for IS*Ecp*1 in 14 isolates.

**Conclusion:**

To the best of our knowledge, this study provides the first molecular characterization of beta-lactamase genes in *E. coli* isolated from the bloodstream in elderly patients. Beta-lactamase genes were detected at a relatively high frequency in elderly patients with bloodstream *E. coli* infections. Plasmid replicon analysis showed that horizontal dissemination of beta-lactamase genes was mainly mediated by IncK and IncF plasmids, which could encode multidrug resistance genes. The study also provides the first report of IS*Aba*1-*bla*_ADC__–__162_-*tnp*A and IS*Ecp*1-*bla*_CTX–M–__14_-IS903-*bla*_CMY–__2_-*blc*-*sug*E in *E. coli*, and demonstrates IncF plasmid-mediated *bla*_ADC__–__162_ and *bla*_CMY–__2_ gene dissemination among bacteria.

## Introduction

*Escherichia coli* is commonly isolated from clinical bloodstream infections. It is referred to as extraintestinal pathogenic *E. coli* (Hung et al., 2019) and is usually multidrug-resistant, potentially leading to sepsis and even death of infected patients (van der Mee-Marquet et al., 2015). Antibiotic selection as a result of the extensive clinical application of broad-spectrum antibiotics, especially third-generation cephalosporins, has led to the generation of drug-resistant bacteria (Baron et al., 2014). Bloodstream infection by multidrug-resistant *E. coli* thus presents difficulties in clinical treatment and has become an important public health problem (Bartoletti et al., 2014). The main resistance mechanism of Gram-negative bacteria such as *E. coli* involves the production of a variety of hydrolytically active beta-lactamases, from broad- to extended-spectrum enzymes, and the enzymatic hydrolysis profile and host range are constantly changing from chromosome-mediated to plasmid-mediated AmpC beta-lactamases (Du et al., 2002; Razazi et al., 2012).

The major beta-lactamase resistance genes in *E. coli* are currently members of the *bla*_CTX__–M_ and *bla*_TEM_ groups, which have been reported in many different countries (Andrea et al., 2013). *bla*_TEM_ were the first beta-lactamase genes found in Gram-negative bacteria. They are specifically transferred by plasmids, and more than 200 subtypes have been identified, mainly encoding enzymes that hydrolyze penicillin and first generation cephalosporins (Medeiros, 1984; Salverda et al., 2010; Clasen et al., 2019). In contrast, *bla*_CTX–M_-encoded enzymes mainly hydrolyze third generation cephalosporins, are mostly located on plasmids of 40–200 kb (Zhao and Hu, 2013), and belong to a wide variety of incompatibility (Inc.) groups. Conjugative plasmids can shuttle between bacteria of the same or different species, thus spreading resistance phenotypes and potentially causing large-scale outbreaks and the prevalence of resistant bacteria. The *bla*_CTX–M_ group beta lactamases have been widely reported to cause bacterial resistance by conjugative plasmids, though the *bla*_CTX_-_M_ group, other *ampC* beta-lactamase resistance genes, such as *bla*_ADC_ and *bla*_CMY_, are also gradually increasing in clinical strains, resulting in greater cephalosporin resistance (Koh et al., 2004). Ceftazidine is the main third generation cephalosporin used in Europe, while cefotaxime and cefoperazone are the most extensively used in China (Lambert et al., 2011). Bacteria with different beta-lactamase genotypes express enzymes with different physical and chemical properties and differences in drug resistance (Medeiros, 1984). Importantly, genotype prevalence also differ among regions. However, the characterization of beta-lactamases in bloodstream-infection *E. coli* from elderly patients in China has not yet been reported, and whether the upstream and downstream surroundings of these genes in bloodstream-infection *E. coli* are consistent with other species of bacteria, or if they have special transfer and transmission capabilities, remains unknown. In this study, we carried out phenotypic and genotypic analyses of bloodstream-infection *E. coli* in a tertiary hospital in China to elucidate the genetic environment in selected isolates in relation to different beta-lactamase types on plasmids in different incompatibility groups. This analysis of the genetic context of the beta-lactamase genes may help to clarify their acquisition, with regard to their origin and further dissemination.

## Materials and Methods

### Bacterial Strains

A total of 1242 *E. coli* isolates were recovered from patient samples at Shanghai Sixth People’s Hospital South Campus, China, during 2015–2018. Among these, 50 strains of bloodstream-infection *E. coli* were isolated from elderly patients and further characterized with regard to extended-spectrum beta-lactamase (ESBL) and *ampC* genes. *E. coli* ATCC25922, J53, and *E. coli* DH5α were maintained in our laboratory.

### Antimicrobial Susceptibility Testing

Antibiotic susceptibility was determined by disk diffusion or broth dilution, using *E. coli* ATCC25922 as a control strain. The tested antibiotics included: amikacin, gentamicin, tobramycin, trimethoprim/sulfamethoxazole, chloramphenicol, meropenem, imipenem, ciprofloxacin, levofloxacin, ampicillin, aztreonam, cefepime, cefotaxime, ceftazidime, cefazolin, ceftriaxone, and cefoxitin. The results were interpreted in accordance with the guidelines of the Clinical and Laboratory Standards Institute (CLSI, 2018).

### Phenotypic Characterization

ESBL production was determined by double-disc synergy tests and confirmed by E-testing, using cefotaxime-cefotaxime-clavulanic acid and ceftazidime-ceftazidime-clavulanic acid strips, according to the CLSI guidelines. Similarly, phenotypic confirmation of plasmid-mediated AmpC production was performed using the AmpC assay *E*-test with cefotetan-ceftatan-clozacillin strips, according to the manufacturer’s instructions (BioMerieux, France).

### Characterization of Beta-Lactamase Genes

The beta-lactamase genotype of each isolate was determined by polymerase chain reaction (PCR) amplification with specific primers for *bla*_TEM_, *bla*_SHV_, *bla*_CTX–M–__1_, *bla*_CTX–M–__2_, *bla*_CTX–M–__8_, *bla*_CTX–M–__9_, *bla*_CTX–M–__25_, *bla*_OXA–__1__,_ *bla*_PER_, *bla*_SME_, *bla*_KPC_, *bla*_VIM_, *bla*_NDM_, *bla*_IMP_, *bla*_GES_, *bla*_VEB_, *bla*_DHA_, *bla*_ADC_, *bla*_ACC_, *bla*_CIT_, and *bla*_EBC_, with bacterially isolated DNA as an amplification template. The total volume of the PCR amplification system was 20 μL, containing 1 μL of genomic DNA template (>50 ng/μL), 10 μL of Premix-rTaq PCR solution (TaKaRa, Japan), 0.4 μL of each primer (10 pmol), and 7 μL of distilled water. PCR was performed using a ProFlex Base Thermal Cycler (Applied Biosystems, Thermo Fisher Scientific, Singapore). The template was denatured at 94°C for 4 min, followed by 35 cycles of 94°C for 40 s, 55°C for 40 s, and 72°C for 40 s, with a final extension stage at 72°C for 5 min. The PCR product was verified by agarose gel electrophoresis and sequencing. All beta-lactamase gene sequencing results were aligned using the BLAST program^1^.

### Conjugation Experiments

Conjugation experiments were performed using sodium azide-resistant *E. coli* J53 as a receptor. Transconjugants were selected on Luria-Bertani agar plates supplemented with sodium azide (200 μg/mL) (Sigma, Germany) and ampicillin (100 μg/mL). J53 and donor bacteria were resuscitated overnight, and a single colony was selected and enriched for 18 h in LB liquid medium without antibiotics. J53 and donor bacteria were mixed 1:4, and a 0.22 μm pore size filter was applied to the blood plate. Thereafter, 150 μL of the mixed bacteria solution was taken up and added to the filter membrane, and cultured overnight. The filter with the bacteria was then removed and washed in LB liquid medium, diluting 1:100 and 150 μL was then applied to the double-antibody plate, cultured overnight, and a single colony was picked for further experiments. The presence of the beta-lactamase gene in the transconjugant was examined using the primers given in Table 1, and the susceptibility of the strain was tested experimentally, as described above.

**TABLE 1:** Primers used for PCR amplification.

**Primer**	**Primer sequence (5′–3′)**	**References**
TEMF	TCGGGGAAATGTGCG	Velasova et al., 2019
TEMR	TGCTTAATCAGTGAGGCACC	Velasova et al., 2019
SHVF	GCCTTTATCGGCCTTCACTCAAG	Velasova et al., 2019
SHVR	TTAGCGTTGCCAGTGCTCGATCA	Velasova et al., 2019
PRE-F	GCTCCGATAATGAAAGCGT	Che et al., 2014
PRE-R	TTCGGCTTGACTCGGCTGA	Che et al., 2014
SME-F	GAGGAAGACTTTGATGGGAGGAT	Che et al., 2014
SME-R	TCCCCTCAGGACCGCCAAG	Che et al., 2014
CTX-M-1F	CAGAGATTTTGCCGTCTAAG	Xiao et al., 2019
CTX-M-1R	GGCCCATGGTTAAAAAATCACTGC	Xiao et al., 2019
CTX-M-2F	CTCAGAGCATTCGCCGCTCA	Xiao et al., 2019
CTX-M-2R	CCGCCGCAGCCAGAATATCC	Xiao et al., 2019
CTX-M-8F	ACTTCAGCCACACGGATTCA	Xiao et al., 2019
CTX-M-8R	CGAGTACGTCACGACGACTT	Xiao et al., 2019
CTX-M-9F	GTTACAGCCCTTCGGCGATGATTC	Xiao et al., 2019
CTX-M-9R	GCGCATGGTGACAAAGAGAGTGCAA	Xiao et al., 2019
CTX-M-25F	GCACGATGACATTCGGG	Xiao et al., 2019
CTX-M-25R	AACCCACGATGTGGGTAGC	Xiao et al., 2019
OXA-1-F	GGCACCAGATTCAACTTTCAAG	Che et al., 2014
OXA-1-R	GACCCCAAGTTTCCTGTAAGTG	Che et al., 2014
ADC-F	GGTATGGCTGTGGGTGTTATTC	This study
ADC-R	CTAAGACTTGGTCGAAAGGT	This study
KPC-F	CGTCTAGTTCTGCTGTCTTG	This study
KPC-R	CTTGTCATCCTTGTTAGGCG	This study
NDM-F	GGTTTGGCGATCTGGTTTTC	This study
NDM-R	CGGAATGGCTCACGATC	This study
IMP-F	GGAATAGAGTGGCTTAAYTCTC	This study
IMP-R	GGTTTAAYAAAACAACCACC	This study
VIM-F	GATGGTGTTTGGTCGCATA	This study
VIM-R	CGAATGCGCAGCACCAG	This study
VEB-F	GCGGTAATTTAACCAGA	This study
VEB-R	GCCTATGAGCCAGTGTT	This study
GES-F	GTTTTGCAATGTGCTCAACG	This study
GES-R	TGCCATAGCCAATAGGCGTAG	This study
DHA-F	AACTTTCACAGGTGTGCTGGGT	This study
DHA-R	CCGTACGCATACTGGCTTTGC	This study
EBC-F	TCGGTAAAGCCGATGTTGCGG	This study
EBC-R	CTTCCACTGCGGCTGCCAGTT	This study
ACC-F	AACAGCCTCAGCAGCCGGTTA	This study
ACC-R	TTCGCCGCAATCATCCCTAGC	This study
CIT-F	TGGCCAGAACTGACAGGCAAA	This study
CIT-R	TTTCTCCTGAACGTGGCTGGC	This study
chuA-F	GACGAACCAACGGTCAGGAT	Clermont et al., 2000
chuA-R	TGCCGCCAGTACCAAAGACA	Clermont et al., 2000
yhaA-F	TGAAGTGTCAGGAGACGCTG	Clermont et al., 2000
yhaA-R	ATGGAGAATGCGTTCCTCAAC	Clermont et al., 2000
TspE4.C2-F	GAGTAATGTCGGGGCATTCA	Clermont et al., 2000
TspE4.C2-R	CGCGCCAACAAAGTATTACG	Clermont et al., 2000
IS26-F	TTACATTTCAAAAACTCTGCTTACC	This study
ISEcp1-F	CAAAATGATCCCCTCGTCAAC	This study
IS903-R	GTTTAATGACCAGCACAGT	This study
ORF477-R	TCGTTTCGTGGTGCTGAATTT	This study
blc-R	TTTAGGTAACGCACGTTGGA	This study
sug-R	GGGCTGTTCTCCTGAATGAT	This study
ISAba1-F	TGGCACTTGCTTAATAAACGTGG	This study
tnpA-F	CATCACCGCGATAAAGCACC	This study
tnpA-R	GGCTCAAAGGCAATACGACC	This study
M9R-F	GAATTGCCTCCTAGTACGCTTAA	This study
M9R-R	CGGTTACGTAAGCTAGCTAGAAT	This study

### Plasmid Replicon Typing

Plasmid DNA was isolated from *E. coli* using a SanPrep Column Plasmid Mini-Preps Kit plasmid isolation system (Sangon Biotech, Shanghai, China) and stored at −20°C, according to the manufacturer’s instructions. Plasmid replicon typing of *E. coli* was performed using a multiplex PCR-based method with 18 pairs of primers, as described previously (Carattoli et al., 2005). The typing results were obtained by agarose gel electrophoresis and verified by sequencing.

### Analysis of Genetic Environment of Beta-Lactamase Genes

**FIGURE 1:**
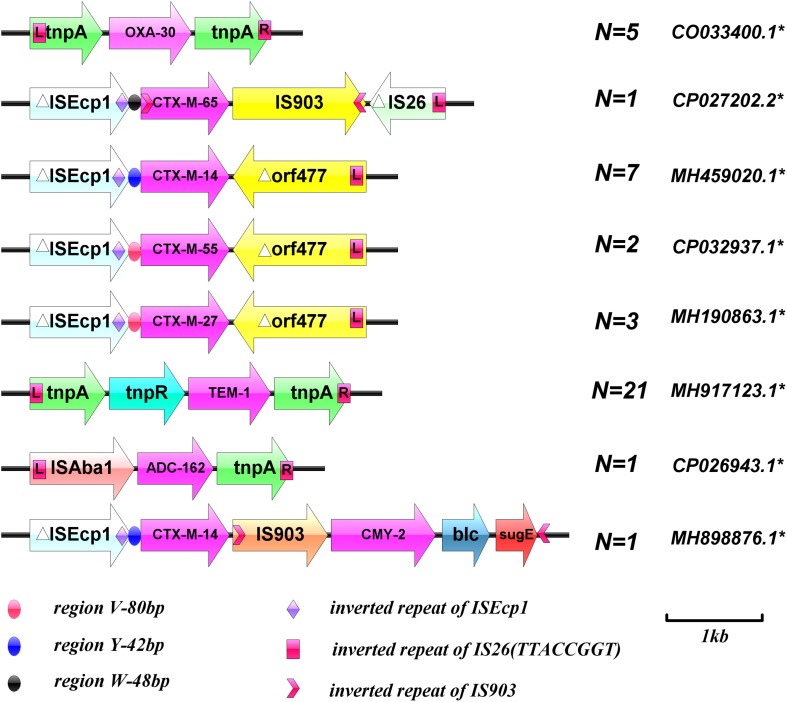
Schematic diagram of the genetic environment surrounding the beta-lactamase genes. ^∗^GenBank Accession numbers. (Sequences of PCR products were analyzed with BLAST to identify target homologous sequences and their GenBank accession numbers; https://blast.ncbi.nlm.nih.gov/Blast.cgi).

The genetic environment of the beta-lactamase genes was detected by long PCR using LA-Taq PCR solution (TaKaRa) according to the manufacturer’s instructions. Primers to detect the genetic environment surrounding the beta-lactamase genes were designed according to the sequences corresponding to the GenBank Accession numbers listed in Figure 1, using Vector NTI advance 11.0 software. The template was initially denatured at 94°C for 4 min, followed by 35 cycles of 94°C for 40 s, 55°C for 1 min, and 72°C for 5 min, with a final extension at 72°C for 10 min. The PCR products were verified by agarose gel electrophoresis and sequencing, and all sequencing results were aligned using the BLAST program. The primers are listed in Table 1.

### Phylogenetic Grouping and Sequence Type (ST) Determination

The major phylogenetic group of each *E. coli* strain was determined by multiplex PCR, using the primers listed in Table 1 (Clermont et al., 2000). Multilocus sequence typing (MLST) was performed according to the Pasteur protocol^2^. Eight conserved housekeeping genes were amplified by PCR using primer sets for *dinB*, *icdA*, *pabB*, *polB*, *putB*, *trpA*, *trpB*, and *uidA*, and sequenced (Table 1). Allele profiles and ST assays were performed according to the *E. coli* MLST website^2^ protocol.

## Results

### Antimicrobial Susceptibility

Fifty bloodstream-infection *E. coli* isolates were obtained from elderly patients (average age, 70.86 years, range 51–92 years; 30% men, 70% women). Most isolates came from the intensive care unit (76%, 38/50), and others from the hematology medical ward (14%, 7/50) and other wards (10%, 5/50). *In vitro* antimicrobial susceptibility testing showed that most isolates were sensitive to gentamicin (32%), trimethoprim/sulfamethoxazole (38%), chloramphenicol (46%), ciprofloxacin (48%), levofloxacin (50%), ampicillin (82%), aztreonam (36%), cefepime (54%), cefotaxime (54%), ceftazidime (24%), cefazolin (54%), ceftriaxone (21%), and cefoxitin (9%). Moreover, all the isolates were sensitive to imipenem, amikacin, tobramycin, and meropenem.

### Genotypes of Beta-Lactamase Genes

Of the 50 strains, 33 were positive for the beta-lactamase genotype [21 ESBL phenotypes/beta-lactamase genotype positive (Table 2); 12 beta-lactamase genotype positive/phenotype negative (Table 3)], Of these 33 strains, one (3%, 1/33) contained three beta lactamase genes, eight (24%, 8/33) contained two beta lactamase genes, and 24 (73%, 24/33) contained only one beta lactamase gene. Among the beta-lactamase-producing strains, 21 isolates were positive for *bla*_TEM_, one for *bla*_SHV_, one for *bla*_CIT,_ two for the *bla*_CTX–M–__1_ group, 13 for the *bla*_CTX–M–__9_ group, and five for the *bla*_OXA–__1_ group. Nucleotide sequence analysis showed that 21 *bla*_TEM_-positive isolates carried *bla*_TEM–__1_ and both *bla*_CTX–M–__1_ group-positive isolates carried *bla*_CTX–M–__55_. Of 13 *bla*_CTX–M–__9_ group-positive isolates, one had *bla*_CTX–M–__65_, three had *bla*_CTX–M–__27_, and nine carried *bla*_CTX–M–__14_. The only *bla*_SHV_ sequenced was *bla*_SHV–__42_, the only *bla*_ADC_ sequenced was *bla*_ADC__–__162_, the only *bla*_CIT_ sequenced was *bla*_CMY–__2_, and all 5 *bla*_OXA–__1_ group-positive isolates carried *bla*_OXA–__30_. Meanwhile, all 50 isolates were negative for *bla*_S__ME_, *bla*_PER_, *bla*_DHA,_ *bla*_ACC,_ *bla*_EBC,_ *bla*_CTX–M–__2_ group, *bla*_CTX–M–__8_ group, and *bla*_CTX–M–__25_ group (Tables 2, 3).

**TABLE 2:** ESBL-positive bloodstream-infection *Escherichia coli* resistance phenotypes and genotypes.

**Strain**	**ESBL**	**MLST**	**PG**	**Plasmid replicon type**	**MIC**						***bla* gene product**	**Transfer**
					**ATM**	**FEP**	**CTX**	**CAZ**	**CZO**	**FOX**		
EC-2	+	ST51	B2	FIA,FIB,FrepB	>16	>16	>32	16	>16	≤	OXA-30	+
EC-4	+	ST9	A	ND	16	16	>32	≤1	>16	16	OXA-30,CTX-M-65	+
EC-6	+	ST45	D	FIA,FIB,FrepB,N,K	≤2	>16	16	≤1	>16	≤8	CTX-M-14	+
EC-7	+	ST48	B2	FIA,FIB,I1,K	>16	>16	>32	8	>16	≤8	TEM-1,CTX-M-55,CTX-M-14	+
EC-9	+	ST95	B2	FIC,K	8	>16	>32	>16	>16	>32	CMY-2,CTX-M-14	+
EC-13	+	ST2	B2	FIA,FIB,FrepB,N,K	>16	>16	>32	>16	>16	≤8	TEM-1	+
EC-20	+	ST730	B2	FrepB,K	4	>16	>32	≤1	>16	≤8	CTX-M-14	+
EC-24	+	ST131	B2	FIB,FrepB,K	8	>16	>32	4	>16	16	CTX-M-27	+
EC-25	+	ST48	B1	FIB,FrepB,K	>16	>16	>32	>16	>16	≤8	TEM-1	+
EC-27	+	ST131	B2	FIA,FIB,FrepB,N,K	>16	>16	>32	>16	>16	≤8	TEM-1,CTX-M-55	+
EC-29	+	ST31	B2	FIA,FIB,FrepB,N,K	≤2	16	>32	≤1	>16	16	TEM-1	+
EC-31	+	ST9	D	FIB,FrepB,K	≤2	16	>32	≤1	>16	≤8	OXA-30,CTX-M-14	+
EC-32	+	ST51	D	I1,Y,K	>16	>16	>32	16	>16	≤8	TEM-1	+
EC-36	+	ST131	B2	FIA,FIB,FrepB,N,K	>16	>16	>32	>16	>16	≤8	CTX-M-27	+
EC-37	+	ST2	B2	FIB,FrepB,K,P	4	>16	>32	≤1	>16	≤8	TEM-1	+
EC-38	+	ST95	A	FIC	8	>16	>32	2	>16	>32	ADC-162	+
EC-39	+	ST681	B2	FIA,FIB,FrepB	>16	>16	>32	2	>16	≤8	TEM-1,CTX-M-14	+
EC-41	+	ST131	B2	FIB,FrepB,K	16	>16	>32	4	>16	16	TEM-1,CTX-M-27	+
EC-43	+	ST48	D	K,B	8	>>16	>32	2	>16	16	TEM-1,CTX-M-14	+
EC-48	+	ST9	B2	FIA,FIB	>16	>16	>32	>16	>16	16	TEM-1	+
EC-50	+	ST9	D	FIB,FrepB,K	≤2	>16	>32	≤1	>16	16	OXA-30,CTX-M-14	+

**PG, phylogenetic group; MIC, minimum inhibitory concentration.**

**TABLE 3:** ESBL-negative bloodstream-infection *Escherichia coli* resistance phenotypes and genotypes.

**Strain**	**ESBL**	**MLST**	**PG**	**Plasmid replicon type**	**MIC**						***bla* gene product**	**Transfer**
					**ATM**	**FEP**	**CTX**	**CAZ**	**CZO**	**FOX**		
EC-1	−	ST51	B2	FIA,FIB,K	≤2	≤2	≤1	≤1	≤4	≤8	TEM-1	+
EC-3	−	ST2	B2	ND	≤2	≤2	≤1	≤1	≤4	≤8	TEM-1	+
EC-16	−	ST1	B1	FrepB,K	≤2	≤2	≤1	≤1	≤4	≤8	TEM-1	+
EC-18	−	ST51	B2	K	≤2	≤2	≤1	≤1	≤4	≤8	TEM-1	+
EC-21	−	ST2	A	FrepB,K	≤2	≤2	≤1	≤1	≤4	≤8	TEM-1	+
EC-23	−	ST8	B2	FIB,FrepB,K	≤2	≤2	≤1	≤1	≤4	≤8	TEM-1	+
EC-26	−	ST2	B2	K	≤2	≤2	≤1	≤1	≤4	≤8	TEM-1	+
EC-28	−	ST117	A	FIB,FrepB,K	≤2	≤2	≤1	≤1	≤4	≤8	TEM-1	+
EC-33	−	ST45	B2	FIB,FrepB,K	≤2	≤2	≤1	≤1	≤4	≤8	TEM-1	+
EC-35	−	ST730	A	ND	≤2	≤2	≤1	≤1	≤4	≤8	OXA-30	−
EC-42	−	ST95	A	ND	≤2	≤2	≤1	≤1	≤4	≤8	SHV-42	+
EC-49	−	ST51	B1	K	≤2	≤2	≤1	≤1	≤4	≤8	TEM-1	+

**PG, phylogenetic group; MIC, minimum inhibitory concentration.**

### Conjugation Experiments

All 50 *E. coli* isolates were tested by conjugation and 39 strains were successfully transferred. Thirty-two of the 33 strains carrying beta-lactamase genes were successfully conjugated, but EC-35 was not successfully conjugated. Cefotaxime- and ceftazidime-resistance phenotypes were simultaneously transferred to sodium azide-resistant *E. coli* J53 recipients by conjugation in beta-lactamase-positive *E. coli* isolates, respectively.

The conjugate was detected by amplifying the beta lactamase gene primer and the results showed that most of the beta lactamase genes were transferred. However, four OXA-1-group (EC-4,EC-31,EC-35,EC-48), three TEM-group (EC-7,EC-32-EC-49), and four CTX-M-9-group (EC-4,EC-7, EC-27,EC-41) genes did not transfer and were not amplified in the corresponding transconjugants. Resistance to non-beta-lactamase antimicrobials was also co-transferred in some cases, in addition to the transfer of extended-spectrum cephalosporin resistance (Clasen et al., 2019). The characteristics of the *E. coli* J53 transconjugants carrying beta-lactamase genes are shown in Table 4.

**TABLE 4:** Genotypes and drug-resistance phenotypes of bloodstream-infection *Escherichia coli* conjugates.

**Transconjugant**	**Donor strain**	**Plasmid replicon type**	***bla* gene product**	**Not detected genotype**	**ESBL**	**Resistance cotransferred**
J-EC-1	EC-1	FIA	TEM-1			AMP^R^
J-EC-2	EC-2	FrepB	OXA-30		+	ATM^R^,FEP^R^,CTX^R^,CZO^R^,CTX^R^
J-EC-3	EC-3	ND	TEM-1			AMP^R^
J-EC-4	EC-4	ND		OXA-30, CTX-M-55	NT	AMP^R^
J-EC-6	EC-6	K	CTX-M-14		NT	
J-EC-7	EC-7	K	CTX-M-14	CTX-M-55, TEM-1	+	ATM^R^,FEP^R^,CTX^R^,CZO^R^,CRO^R^
J-EC-9	EC-9	FIC	CMY-2,CTX-M-14		+	FEP^R^,CTX^R^,CAZ^R^,CZO^R^,CRO^R^,FOX^R^
J-EC-13	EC-13	FrepB	TEM-1		+	ATM^R^,FEP^R^,CTX^R^,CAZ^R^,CZO^R^,CRO^R^
J-EC-16	EC-16	ND	TEM-1			AMP^R^
J-EC-18	EC-18	ND	TEM-1			AMP^R^
J-EC-20	EC-20	FrepB	CTX-M-14		+	FEP^R^,CTX^R^,CZO^R^
J-EC-21	EC-21	ND	TEM-1			AMP^R^
J-EC-23	EC-23	ND	TEM-1			AMP^R^
J-EC-24	EC-24	K	CTX-M-27		NT	
J-EC-25	EC-25	K	TEM-1		NT	AMP^R^
J-EC-26	EC-26	ND	TEM-1			
J-EC-27	EC-27	FrepB	TEM-1	CTX-M-55	+	ATM^R^,FEP^R^,CTX^R^,CAZ^R^,CZO^R^,CRO^R^
J-EC-28	EC-28	ND	TEM-1			AMP^R^
J-EC-29	EC-29	FrepB	TEM-1		NT	
J-EC-31	EC-31	K	CTX-M-14	OXA-30	NT	
J-EC-32	EC-32	I1		TEM-1	NT	
J-EC-33	EC-33	ND	TEM-1			
J-EC-36	EC-36	N	CTX-M-27		NT	
J-EC-37	EC-37	K	TEM-1		+	FEP^R^,CTX^R^
J-EC-38	EC-38	FIC	ADC-162			FEP^R^,CTX^R^,CAZ^R^,CZO^R^,CRO^R^,FOX^R^
J-EC-39	EC-39	K	TEM-1,CTX-M-14		+	ATM^R^,FEP^R^,CTX^R^
J-EC-41	EC-41	FrepB	TEM-1	CTX-M-27	NT	AMP^R^
J-EC-42	EC-42	ND	SHV-42			AMP^R^
J-EC-43	EC-43	FrepB	TEM-1,CTX-M-14		+	FEP^R^,CTX^R^
J-EC-48	EC-48	ND	TEM-1		NT	
J-EC-50	EC-50	K	CTX-M-14	OXA-30	+	FEP^R^,CTX^R^,CZO^R^
J-EC-49	EC-49	ND		TEM-1		AMP^R^

**EC-35 had no transconjugants. ND, Not detected; NT, not transferred, donor strain ESBL phenotype positive but conjugant strain ESBL phenotype negative.**

### Plasmid Analysis

Replicon-typing data for the clinical isolates carrying beta-lactamase genes (Table 2) revealed 10 different replicon types. Among these, two or more plasmid replicon types were detected simultaneously in the 19/21 strain, no plasmid replicon type was detected in the EC-4 strain, and only one plasmid replicon (IncFIC) was carried in the EC-38 strain. The IncF plasmid replicon type was the most common replicon among both types of isolates. Among the ESBL-phenotype-negative strains, plasmid replicon types were not detected in three strains, and the remaining nine strains included only four plasmid replicon types, but all contained IncK plasmid replicons (Table 3).

Plasmid replicon analysis was carried out in the 32 strains with beta-lactamase genes and successful conjugation. Interestingly, no plasmid replicons were detected in 12 transconjugants, and the other 20 transconjugants carried only one plasmid. A total of three plasmid replicon types were detected: IncF (9/19), IncK (9/19), and IncN (1/19) (Table 4). Nine strains were negative for both ESBL genotypes and phenotypes, with no conjugation result and no detection of any plasmid replicon type (Table 5).

**TABLE 5:** ESB-genotype-negative bloodstream-infection *Escherichia coli* resistance phenotypes and characterization of transconjugants.

**Strain**	**MLST**	**PG**	**PRT**	**ESBL**	**MIC**						**Transfer**	**Transconjugants**		
					**ATM**	**FEP**	**CTX**	**CAZ**	**CZO**	**FOX**		**Resistance cotransferred**	**ESBL**	**PRT**
EC-5	ST730	B2	ND	−	≤2	≤2	≤1	≤1	≤4	≤8	−			
EC-8	ST1	B2	ND	+	>16	>16	>32	>16	>16	≤8	+	ATM^R^,FEP^R^, CTX^R^,CAZ^R^, CZO^R^	+	ND
EC-10	ST31	B2	FrepB,K	+	>16	>16	>32	>16	>16	16	+	ATM^R^,CTX^R^, CZO^R^	NT	FrepB
EC-11	ST681	B2	ND	+	>16	≤8	≤2	4	≤8	≤8	−			
EC-12	ST51	B1	ND	−	≤2	≤2	≤1	≤1	≤4	≤8	−			
EC-14	ST51	B2	FrepB,K	+	>16	>16	>32	8	>16	≤8	+	CZO^R^	+	FrepB
EC-15	ST117	B2	ND	−	≤8	≤8	≤2	≤1	≤8	≤8	−			
EC-17	ST45	B1	ND	−	≤2	≤2	≤1	≤1	≤4	≤8	−			
EC-19	ST48	B2	ND	−	≤2	≤2	≤1	≤1	≤4	≤8	−			
EC-22	ST2	A	ND	−	≤2	≤2	≤1	≤1	≤4	≤8	−			
EC-30	ST681	B1	ND	−	≤2	≤2	≤1	≤1	≤4	≤8	−			
EC-34	ST2	D	ND	−	≤8	≤8	≤2	≤1	≤8	≤8	−			
EC-40	ST117	D	FIB,FrepB,I1,K	+	>16	16	>32	>16	>16	≤8	+	CZO^R^	+	FrepB
EC-44	ST2	D	FIB,FrepB,K	+	>16	>16	>32	>16	>16	≤8	+	ATM^R^,FEP^R^, CTX^R^,CZO^R^	+	FrepB
EC-45	ST2	B2	FIA,FIB,K	+	16	>16	>32	4	>16	≤8	+	−	+	ND
EC-46	ST2	D	FrepB,K	−	≤2	≤2	≤1	≤1	≤4	≤8	+	−		FrepB
EC-47	ST45	B2	ND	−	≤2	≤2	≤1	≤1	≤4	≤8	−			

**PG, phylogenetic group; PRT, plasmid replicon type; MIC, minimum inhibitory concentration.**

### Genetic Environment of Beta-Lactamase Genes

The genetic environment surrounding the beta-lactamase genes was verified. Overlapping PCR showed that all *bla*_CTX–M_ groups were IS*Ecp1* upstream and *orf477* or IS903 downstream. Both *bla*_TEM–__1_ and *bla*_OXA–__30_ were located between two transposes and *bla*_ADC–__162_ was located between IS*Aba*1 and *tnpA*. Unexpectedly, the genetic environment surrounding EC-9 *E. coli* beta-lactamase genes was relatively unique; *bla*_CTX–M–__14_ combined with *bla*_CMY–__2_ through an insertion sequence (IS903), to constitute a composite structure of IS*Ecp1*-*bla*_CTX–M–__14_-IS*903-bla*_CMY–__2_-*blc*-*sug*E (Figure 1).

### Phylogenetic Group and ST Designation

Phylogenetic analysis of *E. coli* isolates carrying beta-lactamase genes showed that 24 belonged to virulent groups B2 (*n* = 19) and D (*n* = 5), and nine to non-toxic groups B1 (*n* = 3) and A (*n* = 6). Similarly, analysis of *E. coli* isolates with non-beta-lactamase genes showed that 13 belonged to virulent groups B2 (*n* = 9) and D (*n* = 4), and four belonged to non-toxic groups B1 (*n* = 3) and A (*n* = 1).

MLST analysis identified 12 unique STs among the 50 *E. coli* isolates (Tables 2, 3, 5): ST1 (*n* = 2), ST2 (*n* = 10), ST9 (*n* = 5), ST31 (*n* = 2), ST45 (*n* = 4), ST48 (*n* = 4), ST51 (*n* = 6), ST95 (*n* = 3), ST117 (*n* = 4), ST131 (*n* = 4), ST681 (*n* = 3), and ST730 (*n* = 3) strains.

## Discussion

In the present study, we characterized the ESBL and AmpC phenotypes and genotypes of beta-lactamase-producing *E. coli* blood isolates from patients in China from 2014 to 2018. These results provide the first extensive molecular report of plasmid-mediated ESBL and AmpC beta-lactamase-producing *E. coli* strains isolated from the bloodstream in elderly patients. Of the 50 *E. coli* isolates studied, 28 were positive for ESBL phenotypes, 33 were positive for beta-lactamase genes, 21 strains were positive for both, and 10 were negative for both. Thirteen (61.9%) strains had *bla*_CTX–M_-type ESBL genes and two (9.5%) produced *ampC* genes. *bla*_CTX–M_-type genes were more common than *bla*_OXA_ and *bla*_SHV_, and *ampC* genes (*bla*_ADC_ and *bla*_CMY_) were observed sporadically. A previous study reported that the *bla*_TEM_ and *bla*_SHV_ genes were the most prevalent while the detection frequency of the CTX-M group was low among *E. coli* isolated from China (Quan et al., 2017), however, the current study found a higher prevalence and variety of *bla*_CTX–M_ genes than previously reported (Shi et al., 2015; Zhao et al., 2016). The results of our study suggest that previous studies may have underestimated the frequency of *bla*_CTX–M_ gene transport in *Escherichia coli* samples isolated from blood, which may be related to increased selective pressure of cephalosporins in China.

The 50 *E. coli* isolates in the current study carried a variety of *bla*_CTX–M_ genes (*bla*_CTX–M–__14_, 8/40; *bla*_CTX–M–__27_, 3/40; *bla*_CTX–M–__55_, 2/40; *bla*_CTX–M–__65_, 1/40), and some also contained *bla*_TEM–__1_ (21/40). Although these *bla*_CTX–M_ variants have previously been reported in *E. coli* strains isolated in many countries (Lambert et al., 2011), few studies have detected the *ampC* gene in bloodstream-infection *E. coli* strains in China (Quan et al., 2017), and no previous studies have isolated *E. coli* from blood samples from elderly patients. In particular, we detected and confirmed a case of the *ampC* gene *bla*_ADC–__162_. Related studies to date have only detected the *ampC* gene *bla*_CMY–__2_ in clinical bloodstream infections of *Escherichia coli* in Europe, though genome-wide sequencing confirmed that certain strains of chicken carry *bla*_CMY–__2_ with high homology (Pietsch et al., 2018; Ebmeyer et al., 2019; Seo et al., 2019). To the best of our knowledge, the current study provides the first evidence for *bla*_ADC–__162_ in *Escherichia coli* strains isolated from clinical samples from patients bloodstream infections. Overall, our results indicated that the diversity of ESBL and/or *ampC* genes in *E. coli* strains is increasing, constituting a potential public health problem.

Among the beta-lactamase-genotype-positive strains, most beta-lactamase genes could be transferred to the recipient *E. coli* J53 strain by conjugation. Interestingly, 32 of the 33 beta-lactamase-genotype-positive strains were also carried conjugant plasmids, while EC-35 failed the conjugation test. EC-35 carried the *bla*_OXA–__30_ gene, but no plasmid replicon was detected. Additional plasmid extraction experiments indicated that EC-35 carries a plasmid of approximately 15 kb in length, and the plasmid DNA amplified was positive for *bla*_OXA–__30_ gene. EC-35 was shown to transmit the *bla*_OXA–__30_ gene through a plasmid, consistent with previous reports (Carattoli, 2009). Eight of the remaining 32 conjugant strains partially or totally lost the beta-lactamase gene. No plasmid replicon was detected in J-EC-4 or J-EC-49, and the beta-lactamase gene was lost completely, and J-EC-7, J-EC-31, and J-EC-50 carried *bla*_CTX–M–__14_, while the other beta-lactamase genes were lost. All the plasmid replicons were IncK and other plasmid replicons were lost, suggesting that *bla*_CTX–M–__14_ can be transmitted by the IncK plasmid in bloodstream-infection *E. coli*. Previous reports indicated that *bla*_CTX–M–__14_ was mainly related to the IncK plasmid in Spain and Britain (Carattoli, 2009). However, although both J-EC-6 and J-EC-31 had *bla*_CTX–M–__14_ mediated by the IncK plasmid, the ESBL phenotype of the conjugant strains was lost. The genetic environment around their donor strains was △IS*Ecp*1 (regionY-42 bp) – *bla*_CTX–M–__14_-*orf*477. A 42 bp region with an identical sequence (Y sequence) was found upstream of the start codon of the beta-lactamase gene (Eckert et al., 2006). Regarding J-EC-6 and J-EC-31, amplification of the primer pair (IS*Ecp*1-F and CTX-M-9-R) failed when identical primers were used to validate the genetic environment around *bla*_CTX–M–__14_. Reverse PCR primers (M9R-F and M9R-R) were designed according to CTX-M-9, and sequencing and analysis of the reverse PCR amplification products revealed five and three base mutations in the△IS*Ecp*1 promoter regions of J-EC-6 and J-EC-31, respectively. This may have been due to an increase in the truncated length of IS*Ecp*1, resulting in the loss of promoters in the primer regions and abnormal expression of *bla*_CTX–M–__14_.

In the present study, only EC-9 and EC-38 strains, with *bla*_CMY–__2_ and *bla*_ADC__–__16__2_, respectively, were resistant to fosfomycin. EC-9 carried FIC and K plasmid replicates, and EC-38 carried FIC plasmid replicates. Both strains were ST95 but belonged to different developmental groups, B2 and A, respectively. The genetic environments around *bla*_CMY–__2_ and *bla*_ADC__–__162_ were IS*Ecp*1 (regionY-42bp) – *bla*_CTX–M–__14_-IS903-*bla*_CMY–__2_-*blc*-*sug*E and IS*Aba*1-*bla*_ADC__–__162_-*tnp*A, respectively. IS903 was inserted between two beta-lactamase genes in EC-9 to form a relatively complex tandem structure. Overlapping PCR demonstrated that IS*Ecp*1 (regionY-42bp)-*bla*_CTX–M–__14_-IS903-*bla*_CMY–__2_-*blc*-*sug*E could be completely transferred by conjugation. IS*Aba*1 in IS*Aba1*-*bla*_ADC__–__162_-*tnp*A is a specific insertion sequence found in *Acinetobacter baumannii*, which can mediate the transfer of drug-resistance genes. The current study provides the first evidence for the existence of the same IS*Aba*1-*bla*_ADC__–__162_-*tnp*A insertion sequence in *E. coli*, suggesting that IS*Aba*1 can be transmitted between *E. coli* and *A. baumannii* as a mobile genetic structural element.

Interestingly, *bla*_CMY–__2_ and *bla*_ADC__–__162_ were successfully detected in J-EC-9 and J-EC-38, respectively, and both carried only FIC plasmid replicons, indicating that *bla*_CMY–__2_ and *bla*_ADC__–__162_ were both transported by the IncF plasmid. Although only one IncF plasmid-mediated *bla*_CMY–__2_ and one *bla*_ADC__–__162_-positive strain were detected in this study and no evidence of a cloning epidemic was found, the results suggested the need to remain highly vigilant. The IncF plasmid is known to contain three basic replicates: RepFIA, RepFIB, and RepFIC. The IncF plasmid is a narrow host plasmid with a specific region encoding multidrug-resistance genes. It is only prevalent in Enterobacteriaceae bacteria and can be used as a cloning agent (Yang et al., 2015). Multidrug resistance of the IncF plasmid is closely related to its mobile elements, including the insertion sequence, integron, and transposon, allowing it to capture or recombine resistance genes (Saul et al., 1989; Villa et al., 2010; Yang et al., 2015). It is therefore easy to conjugate and transfer, resulting in the dissemination of *bla*_CMY–__2_ and *bla*_ADC__–__162_ clones, with potentially adverse consequences. To the best of our knowledge, this is the first report of IncF-plasmid-mediated *bla*_CMY–__2_ and *bla*_ADC__–__162_ in bloodstream-infection *E. coli*, indicating the need to be vigilant.

The current results demonstrated that the horizontal transmission of beta-lactamase genes in bloodstream *E. coli* strains is mainly mediated by IncF and IncK plasmids. Despite the differences in plasmid skeleton and variety, few replicons were detected in bloodstream infection *E. coli*, and the conjugates carried only one type of replicon at most. In addition, the plasmids and genetic environment of the *bla*_CTX–M_ group play an important role in regulating the expression, transfer, and transmission of resistance genes. Detection of IS*Ecp*1 upstream of *bla*_CTX–M_, *bla*_CMY–__2_, and *bla*_CMY–M_ genes with different plasmids showed that IS*Ecp*1 plays an important role in capturing, expressing, and continuously mobilizing *bla*_CTX–M_ group and *bla*_CMY–__2_ genes. An IS*Ecp*1 insertion sequence upstream of the gene could result in high levels of expression of *bla*_CTX–M_ resistance genes and carry these resistance genes between chromosomes and plasmids for transfer, leading to spreading among different strains. Inserted sequences such as IS26, IS903, and *orf*477 are also frequently associated with b*la*_CTX–M_ resistance genes. These mobile genetic elements can be distributed randomly upstream and downstream of *bla*_CTX–M_ resistance genes, forming different genomic components in different *bla*_CTX–M_ resistance genes, acting on drug-resistance genes either together or separately, regulating their expression, and mediating their transmission.

## Conclusion

To the best of our knowledge, this study provides the first molecular characterization of beta-lactamase genes from bloodstream isolates of *E. coli* from elderly patients. Beta-lactamase genes, especially *bla*_TEM–__1_, *bla*_CTX–M–__14_, *bla*_OXA–__30_, *bla*_CTX–M–__27_, *bla*_CTX–M–__55_, and *bla*_CTX–M–__65_, were widely prevalent in bloodstream-infection *E. coli* from these patients. Interestingly, *bla*_CMY–__2_ and *bla*_ADC__–__162_ were both transported by IncF plasmids, which are prone to conjugation, indicating the potential for outbreak epidemics related to these genotypes of bloodstream-infection *E. coli*.

## Data Availability Statement

Publicly available datasets were analyzed in this study. This data can be found in GenBank under accession numbers: CO033400.1, CP027202.2, MH459020.1, CP032937.1, MH190863.1, MH917123.1, CP026943.1, and MH898876.1.

## Ethics Statement

This study used strains obtained from patient blood. The Ethics Committee of Shanghai University of Medicine & Health Sciences Affiliated Sixth People’s Hospital South Campus waived the need for the study to be reviewed or approved by an ethics committee because none of the strains were cultured in primary culture, and no information could be traced directly to any individual patient.

## Author Contributions

LX and QW conceived the study, analyzed the data, and wrote the manuscript. WL coordinated the study. LX, XW, NK, MC, LZ, and MS performed the experiments. LX, QW, and WL revised the manuscript.

## Conflict of Interest

The authors declare that the research was conducted in the absence of any commercial or financial relationships that could be construed as a potential conflict of interest.

## Footnotes

1 http://www.ncbi.nlm.nih.gov/BLAST

2 https://bigsdb.pasteur.fr/ecoli/

## References

[B1] Andrea D.Mm Arena F Pallecchi L. Rossolini G. M. (2013). CTX-M-type β-lactamases: a successful story of antibiotic resistance. *Int. J. Med. Microbiol* 303 305–317. 10.1016/j.ijmm.2013.02.008 23490927

[B3] Baron S. Jouy E. Larvor E. Eono F. Bougeard S. Kempf I. (2014). Impact of third-generation-cephalosporin administration in hatcheries on fecal *escherichia coli* antimicrobial resistance in broilers and layers. *Antimicrob, Agents Chemother.* 58 5428–5434. 10.1128/AAC.03106-14 24982086 PMC4135878

[B4] Bartoletti M. Giannella M. Caraceni P. Domenicali M. Ambretti S. Tedeschi S. (2014). Epidemiology and outcomes of bloodstream infection in patients with cirrhosis. *J. Hepatol.* 61 51–58. 10.1016/j.jhep.2014.03.021 24681345

[B5] Carattoli A. (2009). Resistance plasmid families in Enterobacteriaceae. *Antimicrob. Agents Chemother.* 53 2227–2238. 10.1128/aac.01707-08 19307361 PMC2687249

[B6] Carattoli A. Bertini A. Villa L. Falbo V. Hopkins K. L. Threlfall E. J. (2005). Identification of plasmids by PCR-based replicon typing. *J. Microbiol. Meth.* 63 219–228. 10.1016/j.mimet.2005.03.018 15935499

[B7] Che T. Bethel C. R. Pusztai-Carey M. Bonomo R. A. Carey P. R. (2014). The different inhibition mechanisms of OXA-1 and OXA-24 beta-lactamases are determined by the stability of active site carboxylated lysine. *J. Biol. Chem.* 289 6152–6164. 10.1074/jbc.M113.533562 24443569 PMC3937681

[B8] Clasen J. Birkegard A. C. Graesboll K. Folkesson A. (2019). The evolution of TEM-1 extended-spectrum beta-lactamases in E. coli by cephalosporins. *J. Glob. Antimicrob. Resist.* 19 32–39. 10.1016/j.jgar.2019.03.010 31048029

[B9] Clermont O. Bonacorsi S. Bingen E. (2000). Rapid and simple determination of the *Escherichia coli* phylogenetic group. *Appl. Environ. Microbiol.* 66 4555–4558. 10.1128/aem.66.10.4555-4558.2000 11010916 PMC92342

[B10] CLSI (2018). *Performance Standards for Antimicrobial Susceptibility Testing. Informational Supplement M100-s28*, 28th Edn Wayne, PA: Clinical Laboratory Standards Institute.

[B11] Du B. Long Y. Liu H. Chen D. Liu D. Xu Y. (2002). Extended-spectrum beta-lactamase-producing *Escherichia coli* and *Klebsiella pneumoniae* bloodstream infection: risk factors and clinical outcome. *Intens. Care Med.* 28 1718–1723. 10.1007/s00134-002-1521-1 12447513

[B12] Ebmeyer S. Kristiansson E. Larsson D. G. J. (2019). CMY-1/MOX-family AmpC β-lactamases MOX-1, MOX-2 and MOX-9 were mobilized independently from three aeromonas species. *J. Antimicrob. Chemoth.* 74 1202–1206. 10.1093/jac/dkz025 30753583 PMC6477974

[B13] Eckert C. Gautier V. Arlet G. (2006). DNA sequence analysis of the genetic environment of various blaCTX-M genes. *J. Antimicrob. Chemoth.* 57 14–23. 10.1093/jac/dki398 16291869

[B14] Hung W. T. Cheng M. F. Tseng F. C. Chen Y. S. Shin-Jung L. S. Chang T. H. (2019). Bloodstream infection with extended-spectrum beta-lactamase-producing *Escherichia coli*: the role of virulence genes. *J. Microbiol. Immunol. Infect.* 10.1016/j.jmii.2019.03.005 [Epub ahead of print]. 31076319

[B15] Koh T. H. Wang G. C. Sng L. H. Koh T. Y. (2004). CTX-M and plasmid-mediated AmpC-producing *Enterobacteriaceae* Singapore. *Emerg. Infect. Dis* 10 1172–1174. 10.3201/eid1006.030726 15224678 PMC3323164

[B16] Lambert M. L. Suetens C. Savey A. Palomar M. Hiesmayr M. Morales I. (2011). Clinical outcomes of health-care-associated infections and antimicrobial resistance in patients admitted to European intensive-care units: a cohort study. *Lancet Infect. Dis.* 11 30–38. 10.1016/S1473-3099(10)70258-9 21126917

[B17] Medeiros A. A. (1984). Beta-lactamases. *Br. Med. Bull.* 40 18–27. 6100076 10.1093/oxfordjournals.bmb.a071942

[B18] Pietsch M. Irrgang A. Roschanski N. Brenner Michael G. Hamprecht A. Rieber H. (2018). Whole genome analyses of CMY-2-producing *Escherichia coli* isolates from humans, animals and food in Germany. *BMC Genomics* 19:601. 10.1186/s12864-018-4976-3 30092762 PMC6085623

[B19] Quan J. Zhao D. Liu L. Chen Y. Zhou J. Jiang Y. (2017). High prevalence of ESBL-producing *Escherichia coli* and *Klebsiella pneumoniae* in community-onset bloodstream infections in China. *J. Antimicrob. Chemother.* 72 273–280. 27624571 10.1093/jac/dkw372

[B20] Razazi K. Derde L. P. G. Verachten M. Legrand P. Lesprit P. Brun-Buisson C. (2012). Clinical impact and risk factors for colonization with extended-spectrum β-lactamase-producing bacteria in the intensive care unit. *Intens. Care Med.* 38 1769–1778. 10.1007/s00134-012-2675-0 22893223

[B21] Salverda M. L. De Visser J. A. Barlow M. (2010). Natural evolution of TEM-1 beta-lactamase: experimental reconstruction and clinical relevance. *FEMS Microbiol. Rev.* 34 1015–1036. 10.1111/j.1574-6976.2010.00222.x 20412308

[B22] Saul D. Spiers A. J. McAnulty J. Gibbs M. G. Bergquist P. L. Hill D. F. (1989). Nucleotide sequence and replication characteristics of RepFIB, a basic replicon of IncF plasmids. *J. Bacteriol.* 171 2697–2707. 10.1128/jb.171.5.2697-2707.1989 2651415 PMC209954

[B23] Seo K. W. Shim J. B. Lee Y. J. (2019). Emergence of CMY-2-Producing *Escherichia coli* in Korean layer parent stock. *Microb. Drug Resist.* 25 462–468. 10.1089/mdr.2018.0254 30625027

[B24] Shi H. Sun F. Chen J. Ou Q. Feng W. Yong X. (2015). Epidemiology of CTX-M-type extended-spectrum beta-lactamase (ESBL)-producing nosocomial -*Escherichia coli* infection in China. *Ann. Clin. Microbiol. Antimicrob.* 14:4. 10.1186/s12941-015-0063-7 25591816 PMC4299296

[B25] van der Mee-Marquet N. L. Blanc D. S. Gbaguidi-Haore H. Dos Santos Borges S. Viboud Q. Bertrand X. (2015). Marked increase in incidence for bloodstream infections due to *Escherichia coli*, a side effect of previous antibiotic therapy in the elderly. *Front. Microbiol.* 6:646. 10.3389/fmicb.2015.00646 26175721 PMC4485226

[B26] Velasova M. Smith R. P. Lemma F. Horton R. A. Duggett N. Evans J. (2019). Detection of extended spectrum beta-lactam (ESBL), AmpC and carbapenem resistance in *Enterobacteriaceae* in beef cattle in Great Britain in 2015. *J. Appl. Microbiol.* 126 1081–1095. 10.1111/jam.14211 30693606

[B27] Villa L. Garcia-Fernandez A. Fortini D. Carattoli A. (2010). Replicon sequence typing of IncF plasmids carrying virulence and resistance determinants. *J. Antimicrob. Chemother.* 65 2518–2529. 10.1093/jac/dkq347 20935300

[B28] Xiao L. Wang X. Kong N. Cao M. Zhang L. Wei Q. (2019). Polymorphisms of gene cassette promoters of the class 1 integron in clinical proteus isolates. *Front. Microbiol.* 10:790. 10.3389/fmicb.2019.00790 31068909 PMC6491665

[B29] Yang Q. E. Sun J. Li L. Deng H. Liu B. T. Fang L. X. (2015). IncF plasmid diversity in multi-drug resistant *Escherichia coli* strains from animals in China. *Front. Microbiol.* 6:964 10.3389/fmicb.2015.00964 PMC4585273 26441898

[B30] Zhao S. Y. Zhang J. Zhang Y. L. Wang Y. C. Xiao S. Z. Gu F. F. (2016). Epidemiology and risk factors for faecal extended-spectrum beta-lactamase-producing *Enterobacteriaceae* (ESBL-E) carriage derived from residents of seven nursing homes in western Shanghai. *China. Epidemiol. Infect.* 144 695–702. 10.1017/S0950268815001879 26260355

[B31] Zhao W. H. Hu Z. Q. (2013). Epidemiology and genetics of CTX-M extended-spectrum beta-lactamases in gram-negative bacteria. *Crit. Rev. Microbiol* 39 79–101. 10.3109/1040841X.2012.691460 22697133 PMC4086240

